# Soluble ST2 Plasma Concentrations Predict Mortality in HBV-Related Acute-on-Chronic Liver Failure

**DOI:** 10.1155/2015/535938

**Published:** 2015-03-29

**Authors:** Ziying Lei, Zhishuo Mo, Jianyun Zhu, Xiuqing Pang, Xingrong Zheng, Zhebin Wu, Ke Wang, Xinhua Li, Dongying Xie, Zhiliang Gao

**Affiliations:** ^1^Department of Infectious Disease, The Third Affiliated Hospital of Sun-Yet-Sen University, Guangzhou, Guangdong 510630, China; ^2^Key Laboratory of Tropical Disease Control, Sun Yat-Sen University, Ministry of Education, Guangzhou, Guangdong 510080, China

## Abstract

Hepatitis B virus-related acute-on-chronic liver failure (HBV-ACLF) is a rapidly progressing and frequently fatal condition. The aim of this study was to determine whether interleukin- (IL-) 33 and soluble ST2 (sST2) were associated with disease severity and mortality in HBV-ACLF. We found that plasma levels of sST2 but not IL-33 were higher in HBV-ACLF patients compared with chronic hepatitis B (CHB) patients and healthy controls. However, plasma levels of IL-33, TNF-*α*, IFN-*γ*, and IL-10 did not correlate with sST2 levels. Similarly, immunohistochemistry revealed low IL-33 expression and high ST2 expression in liver sections of patients with HBV-ACLF. Evaluation of dynamic changes of sST2 in HBV-ACLF showed that plasma sST2 levels increased over time in patients who died during the 180-day follow-up but decreased in those who survived. In addition, plasma sST2 level after week 1 correlated with disease severity, as assessed by total bilirubin, prothrombin time, and model for end-stage liver disease score. Results of Kaplan-Meier survival analysis showed that higher sST2 concentration (≥87 ng/mL) at week 3 was associated with poor survival. These findings indicate the potential usefulness of sST2 as a predictor of disease severity and in making treatment decisions for patients with HBV-ACLF.

## 1. Introduction

Acute-on-chronic liver failure (ACLF) refers to an acute hepatic insult in a patient with chronic liver disease that manifests as jaundice and coagulopathy and is complicated within 4 weeks by ascites and/or encephalopathy. The underlying chronic liver diseases in ACLF vary according to geographic region; alcoholic cirrhosis constitutes 50–70% of all the underlying liver diseases of ACLF in the western countries, whereas hepatitis B constitutes 70% of the etiologies of ACLF in most Asian countries. The acute episodes, including both infectious and noninfectious causes, also differ between the East and West. In Asia the acute insult often involves reactivation of the hepatitis B virus (HBV) infection due to intensive chemotherapy, immunosuppressive therapy, or withdrawal of antiviral therapy [1–4].

HBV-ACLF is associated with a mortality rate of 60% to 80% [5, 6]. Supportive measures are provided to all patients, and antiviral therapy with nucleoside/nucleotide analogues is recommended when HBV DNA is detected. Liver transplantation is the definitive treatment for patients who do not improve with supportive measures [4]. However, most patients die because of lack of money for the procedure, lack of organs available for transplantation, or development of serious complications while on the transplant waiting list [7]. Therefore, it is important to predict the prognosis of HBV-ACLF to make appropriate treatment decisions in a timely manner. The model for end-stage liver disease (MELD) scoring system has been shown to be a useful predictor of survival in ACLF patients [8].

Mechanisms underlying the pathogenesis of HBV-ACLF are complicated and remain unclear. However, cytokines including tumor necrosis factor (TNF)-*α*, interferon (IFN)-*γ*, interleukin- (IL-) 1, IL-6, and IL-10 are believed to be involved in the development of liver failure [9–11]. A previous study demonstrated that an imbalance of T helper (Th) 1 and Th2 cytokines was associated with the pathogenesis of liver failure [12]. In addition, IL-33, a newly described member of the IL-1 family, has recently been implicated in ACLF [13]. This cytokine is expressed primarily in the nucleus of epithelial and endothelial cells, where it appears to be involved in regulating transcription [14]. IL-33 is secreted by necrotic cells after tissue damage occurs but is largely retained inside the apoptotic bodies during apoptosis [15]. Therefore, IL-33 is considered an alarmin (i.e., endogenous molecule that signals tissue damage and activates the immune system) [14, 15].

The role of IL-33 in certain autoimmune diseases, allergic diseases, and cardiovascular disease has been confirmed, but its effect can be either pro- or anti-inflammatory, depending on the disease and model of disease [16–20]. The receptor for IL-33 is ST2, an IL-1 receptor family member with transmembrane and soluble isoforms [21]. Transmembrane ST2 (ST2L) is expressed primarily on the membranes of Th2 cells and mast cells and is reported to play a role in T cell-mediated diseases [22]. However, soluble ST2 (sST2), a decoy receptor for IL-33, downregulates the IL-33/ST2 signaling pathway and serves as a cardiovascular biomarker for the presence of ventricular biomechanical overload [23–25].

Although the IL-33/ST2 signaling had been shown to protect against Concanavalin A-induced liver injury [26], its function in HBV-ACLF is unclear. However, immunologic defects with severe neutrophil dysfunction due to endotoxins have been observed in ACLF and are comparable with those seen in sepsis [27]. Hoogerwerf et al. reported recently that sepsis results in sustained elevation of serum sST2, and the sST2 levels correlate with disease severity and mortality [28]. The potential role of IL-33 and sST2 in regulation of the immune response during HBV-ACLF led us to investigate the dynamic changes of plasma IL-33 and sST2 levels in HBV-ACLF patients. We also evaluated the relationship between IL-33 and sST2 and disease severity and mortality.

## 2. Materials and Methods

### 2.1. Patients and Design

This study was approved by the scientific and ethics committees of the Third Affiliated Hospital of Sun Yat-sen University. Written informed consent was obtained from all subjects or their relatives. We followed 48 patients with HBV-ACLF who were admitted to the Third Affiliated Hospital of Sun Yat-sen University between September 2012 and March 2013. All the patients met the diagnostic criteria for HBV-ACLF [4]: positive for the surface antigen of HBV (HBsAg) or detectable HBV DNA for at least 6 months; acute hepatic insult manifesting as jaundice and coagulopathy, complicated within 4 weeks by ascites and/or encephalopathy; total bilirubin (TBIL) ≥85 *μ*mol/L; and prothrombin time international normalized ratio (INR) ≥1.5. We recruited 12 patients with chronic hepatitis B (CHB) from outpatient clinics and 16 healthy individuals with no prior history of liver disease from the physical examination center to serve as controls. We excluded individuals with severe heart failure, sepsis, asthma or anaphylaxis, current hepatitis virus (A, C, D, E) or human immunodeficiency virus (HIV) infection, metabolic or drug-induced liver disease, or uncontrolled autoimmune diseases and those who had received immunosuppressive therapy or died within three weeks since hospital admission.

Patients with HBV-ACLF were given supportive treatments, including antiviral treatment, according to the diagnostic and treatment guidelines for liver failure (2012 version, China) [29]. Blood was obtained once per week using ethylenediaminetetraacetic acid (EDTA) tubes starting at the time of inclusion (week 0) until week 3. After centrifugation, the plasma was stored at −80°C until analysis. All the patients were followed up for 180 days after week 3, and none of these patients underwent liver transplantation. At the end of the follow-up period, 35 patients were alive and 13 had died.

### 2.2. Liver Biopsies

Liver tissue specimens were obtained from 18 patients with HBV-ACLF who underwent liver transplantation in the Third Affiliated Hospital of Sun Yat-sen University from May 2013 to March 2014. In addition, we obtained 16 tissue samples from liver biopsies of CHB patients and 18 tissue samples from healthy donor livers in liver transplantations in the same hospital to serve as controls. These tissue specimens were obtained in accordance with the relevant laws and the requirements of the local Ethics Committee.

### 2.3. Cytokine Detection by Enzyme-Linked Immunosorbent Assay or Luminex xMAP

Plasma levels of sST2 were determined by enzyme-linked immunosorbent assay (ELISA) using commercially available kits (R&D Systems; Minneapolis, MN, USA). Plasma samples from the 48 HBV-ACLF patients, 12 CHB controls, and 16 healthy controls were diluted 100-fold, 40-fold, and 20-fold, respectively, according to the manufacturer's instruction and results of preliminary experiments. Plasma levels of IL-33, TNF-*α*, IFN-*γ*, and IL-10 were determined simultaneously using the Luminex xMAP multiplex system with the Milliplex MAP human Th17 magnetic bead panel kit (Millipore Corporation, Billerica, MA, USA) [30, 31]. The assays were performed on a Bio-Plex 200 system (Bio-Rad, Hercules, CA, USA). Each sample was measured in duplicate. Detection limits were 5 pg/mL (IL-33), 3 pg/mL (TNF-*α*), 10 pg/mL (IFN-*γ*), and 1 pg/mL (IL-10).

### 2.4. Immunohistochemistry

Immunohistochemistry was carried out using standard techniques. Paraffin-embedded tissue blocks were cut into 3.5 *μ*m sections. After dewaxing and rehydration, the sections were incubated with 3% H_2_O_2_ for 10 minutes to inactivate endogenous peroxidases. Antigen retrieval was performed with EDTA (pH 8.0) in the boiling water of a pressure cooker for 4 minutes. The tissue sections were cooled for 20 minutes at room temperature and then incubated for 1 hour at 37°C with primary antibodies against IL33 (1 : 250, rabbit IgG; Sigma-Aldrich, St. Louis, MO, USA) and ST2 (also called IL1RL1) (1 : 20, rabbit IgG; Sigma-Aldrich), which had been diluted with 1% bovine serum albumin. Then the sections were washed with phosphate buffered saline and incubated with the corresponding secondary and thirdly antibodies (NovoLink Polymer Detection System RE7140-CN, Novocastra Laboratories, Newcastle upon Tyne, UK) for 15 minutes at 37°C successively. Slides were developed with the substrate/chromogen 3,3′-diaminobenzidine, counterstained with modified Mayer's hematoxylin, blued in 0.3% ammonia water, and mounted in a routine manner. The tissue sections were viewed using a light microscope (Leica Corporation, Wetzlar, Hesse, Germany), and IL-33^+^ cells were counted.

### 2.5. Clinical Data

Clinical data were collected from patient records and the hospital information system. Serum alanine aminotransferase (ALT), TBIL, and serum creatinine were detected using an automated clinical biochemistry analyzer (Olympus, Japan), and INR was determined by an automatic coagulation analyzer (Diagnostica Stago, France). HBV-related serologies were determined by chemiluminescent microparticle immunoassay using an automated chemiluminescence immunoassay analyzer (Abbott I 2000, USA). Serum HBV DNA was determined by real-time quantitative polymerase chain reaction (ABI 5700, USA). The detection limit was 100 IU/mL.

The mathematical formula for MELD is MELD score = 3.8 × ln⁡⁡ (TBIL × 0.058) + 11.2 × ln⁡⁡ (INR) + 9.6 × ln⁡⁡ (creatinine × 0.011) + 6.4 × (0 for cholestasis or alcoholic liver cirrhosis, 1 for other causes) [8].

### 2.6. Statistical Analysis

All data analyses were carried out using SPSS 13.0. Continuous data were tested for normal distribution and presented as mean ± standard deviation (SD) or median and interquartile range (IQR), as appropriate. Quantitative data were compared by analysis of variance or nonparametric test, and changes in cytokine levels over time were compared by repeated measures analysis of variance. Relationships between any two variables were evaluated by Spearman's rank correlation test. Receiver operating characteristics (ROC) curves and Kaplan-Meier survival analysis were used to determine the predictive value of sST2 for survival of patients with HBV-ACLF.

## 3. Results

### 3.1. Low Levels of IL-33 and High Levels of sST2 in Patients with HBV-ACLF at Baseline

**Figure 1:**
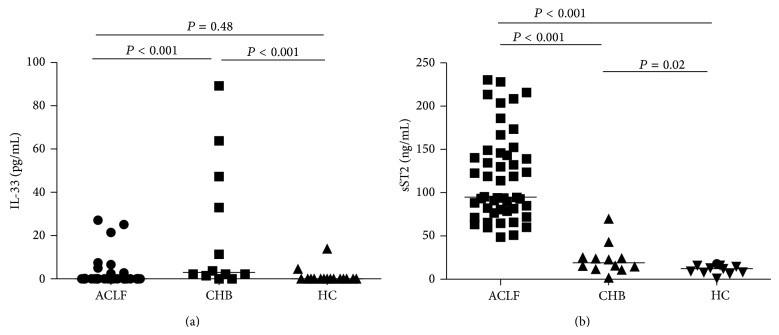
Baseline plasma levels of IL-33 and soluble ST2 (sST2). Individual data points represent mean values of patients with hepatitis B-related acute-on-chronic liver failure (HBV-ACLF,  *n* = 48), patients with chronic hepatitis B (CHB,  *n* = 12), and healthy controls (HC,  *n* = 16). Horizontal lines indicate median values of each group. (a) Baseline plasma levels of IL-33 were determined using Luminex xMAP technology. (b) Baseline plasma sST2 levels were determined by ELISA. Differences between two groups were evaluated using the Mann-Whitney *U* test.

Demographic and clinical characteristics of HBV-ACLF patients and controls are presented in Table 1. We found that IL-33 was detectable in a smaller proportion of patients with HBV-ACLF (12/48, 25%) than patients with CHB (10/12, 83%; *P* = 0.001); however, detection of plasma IL-33 did not differ between the HBV-ACLF group and healthy controls (2/16, 12.5%; *P* = 0.48) (Figure 1(a)). In contrast, median plasma levels of sST2 were significantly higher in the HBV-ACLF group (94.90 ng/mL) compared with the CHB group (19.02 ng/mL) and healthy controls (9.22 ng/mL) (*P* < 0.001) (Figure 1(b)). Baseline levels of sST2 and IL-33 were not correlated.

### 3.2. IL-33 and ST2 Expressions in Human Liver

**Figure 2:**
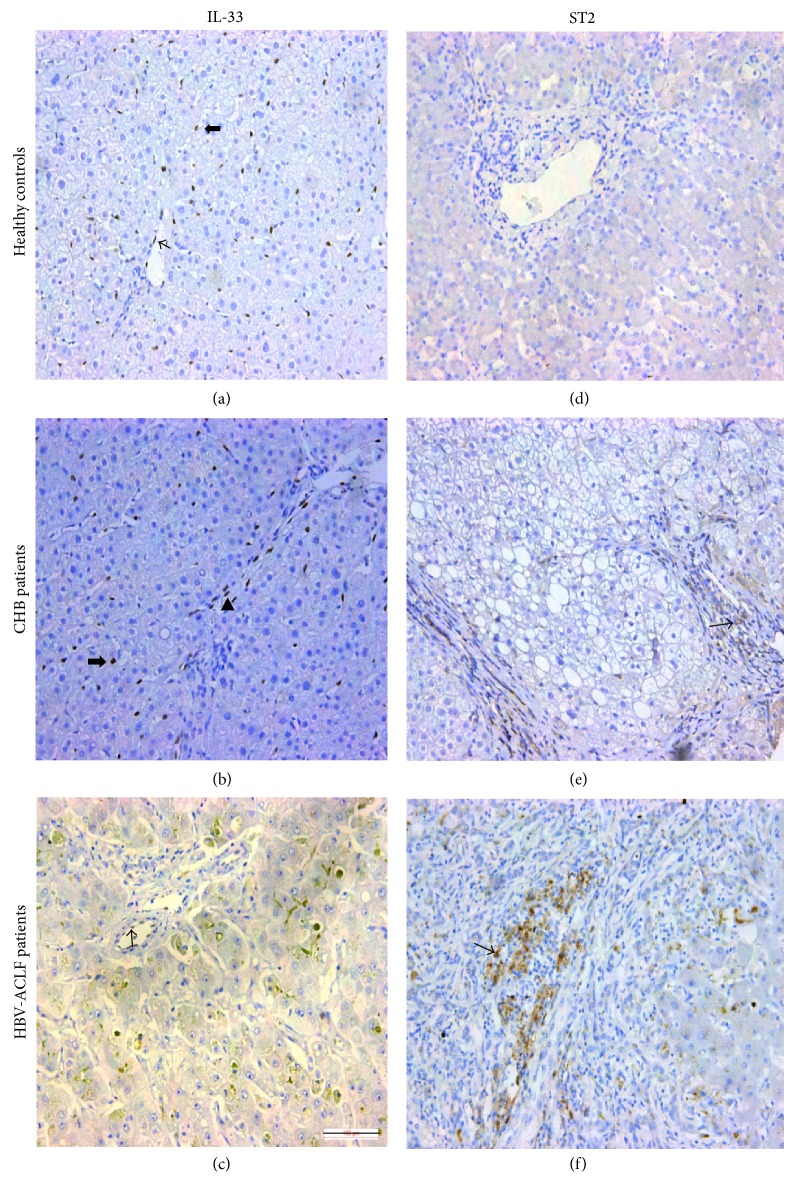
Expression of IL-33 and ST2 in liver specimens of hepatitis B virus-related acute-on-chronic liver failure (HBV-ACLF) patients, chronic hepatitis B (CHB) patients, and healthy controls. Immunohistochemical staining was performed with anti-IL33 (a–c) or anti-ST2 protein (d–f) antibodies. Cells that stained positive for IL-33 are indicated with narrow arrows (endothelial cells) (a, c), wide arrows (sinusoidal cells) (a, b), or arrowhead (fibrous scar cells) (b). Narrow arrows in e and f indicate infiltrating ST2^+^ cells. Scale bar = 100 *μ*m.

**Figure 3:**
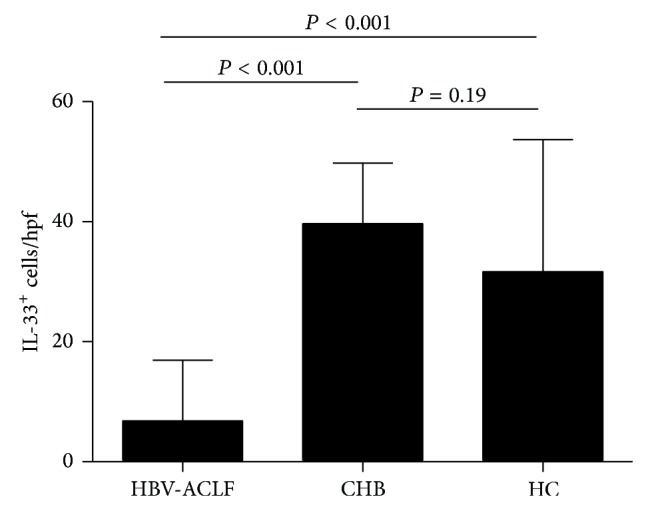
Significantly fewer IL-33^+^ cells were detected in liver tissues obtained from hepatitis B virus-related acute-on-chronic liver failure (HBV-ACLF) patients compared with those obtained from chronic hepatitis B (CHB) patients and healthy controls (HC). Data shown are the mean number of IL-33^+^ cells (±SD) determined in 10 high-power fields (hpf; 200x). Group differences were analyzed by Mann-Whitney *U* test.

To compare expression of IL-33 and ST2 in liver tissues with varying degrees of inflammation, we used immunohistochemistry to detect these proteins in liver tissue specimens obtained from HBV-ACLF patients, CHB patients, and healthy controls (Figure 2). Similar to previous reports [32, 33], IL-33 staining was concentrated in vascular endothelial cells and sinusoid endothelial cells, areas of inflammation, and fibrous scars, with a nuclear localization. In contrast, IL-33 was not detected in biliary ducts or hepatocytes. Significantly fewer IL-33^+^ cells were detected in liver specimens of HBV-ACLF patients compared with those of CHB patients (*P* < 0.001) and healthy controls (*P* < 0.001) (Figure 3); IL-33 was not detected in seven HBV-ACLF patients, and ≤10 IL-33^+^ cells per high-power field were detected in eight HBV-ACLF patients. The number of IL-33^+^ cells did not differ significantly between CHB patients and healthy controls.

In contrast, ST2^+^ cells were not detected on normal liver tissue but were expressed in portal inflammatory infiltrate and fibrous scars in liver sections from 10 CHB patients (62.5%) and all HBV-ACLF patients, with cytoplasmic localization (Figure 2). Staining was stronger and more extensive in HBV-ACLF patients than in CHB patients (data not shown).

### 3.3. Dynamic Changes in Plasma Levels of sST2, IL-33, IL-10, TNF-*α*, and IFN-*γ* in Patients with HBV-ACLF Who Died during Follow-Up versus Those Who Survived

**Figure 4:**
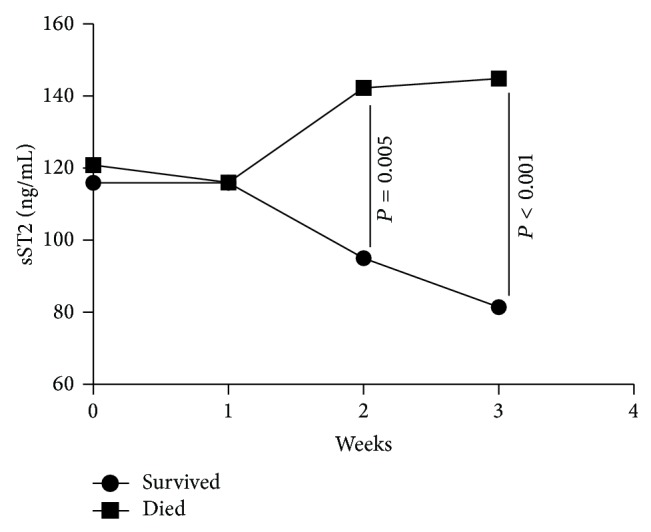
Dynamic changes in mean plasma levels of soluble ST2 (sST2) in patients who survived (*n* = 35) during the 180-day follow-up versus those who died (*n* = 13). Differences between the two groups at a single time point were evaluated using the Mann-Whitney *U* test. Results of repeated measures analysis of variance confirmed that dynamic changes in sST2 levels of the two groups differed (*P* = 0.02).

**Figure 5:**
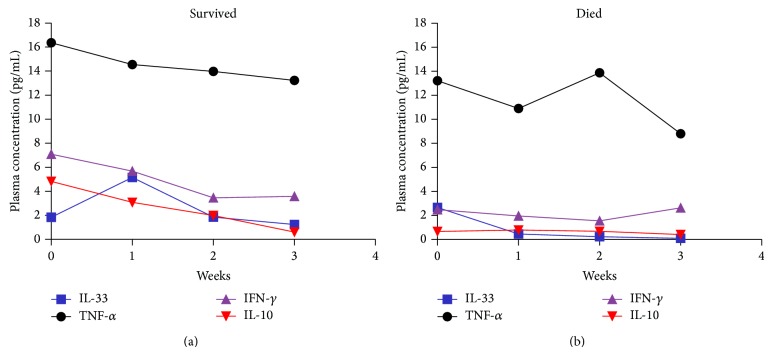
The dynamic process of cytokines in (a) patients who survived during the 180-day follow-up (*n* = 35) and (b) those who died (*n* = 13). Plasma levels of IL-33, TNF-*α*, IFN-*γ*, and IL-10 were simultaneously determined by Luminex xMAP. Data are expressed as mean values; 95% confidence intervals are not shown in the figure for reasons of clarity.

Demographic and clinical characteristics of HBV-ACLF patients who survived during the 180-day follow-up and those who died are presented in Table 2. The level of ALT, TBIL, and INR, except for HBV DNA differed between two groups. Baseline plasma sST2 levels (week 0) did not differ significantly between patients who died versus those who survived. However, plasma sST2 levels increased progressively from week 1 to week 3 in patients who eventually died but decreased over time in those who survived (Figure 4). Results of repeated measure analysis of variance confirmed that dynamic changes in sST2 plasma concentration differed between two groups (*P* = 0.02). The level of IL-33 also decreased over time and was detectable in only six patients (5 survived, 1 died) in week 3 (Figure 5).

To evaluate the relationships between plasma sST2 and levels of pro- or anti-inflammatory mediators in patients with HBV-ACLF, we measured plasma concentrations of TNF-*α*, IFN-*γ*, and IL-10 over time (Figure 5). The mean plasma level of IL-10 at baseline was higher in patients who survived compared with those who died during follow-up (*P* = 0.006) but decreased over time and by week 3 was similar to that of patients who eventually died. Dynamic changes in IL-33, TNF-*α*, IFN-*γ*, and IL-10 did not differ significantly between patients who survived versus those who died, and no correlation was found between cytokine levels and sST2 level at any time point.

### 3.4. Relationship between Plasma sST2 Level and Disease Severity

**Figure 6:**
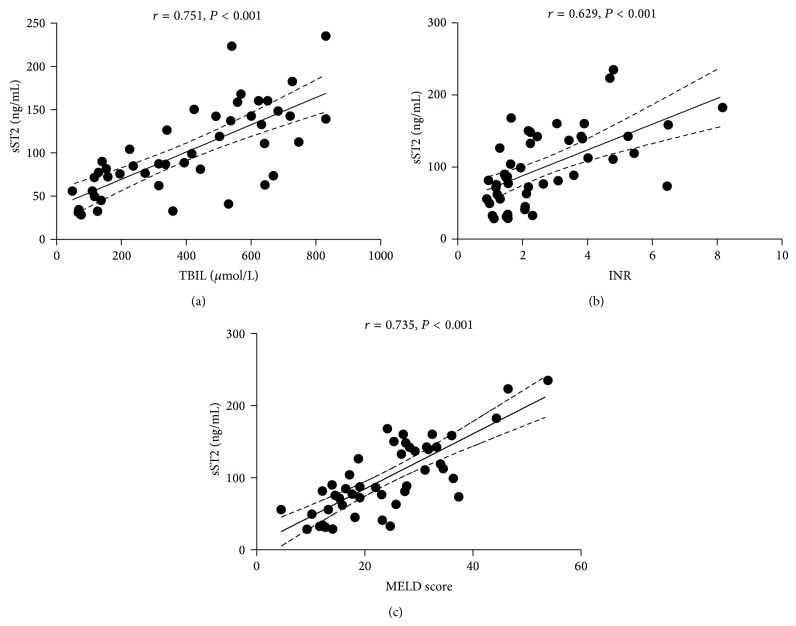
Correlations between soluble ST2 (sST2) and total bilirubin (TBIL), prothrombin time international normalized ratio (INR), and model for end-stage liver disease (MELD) score in week 3. Graphic representation of correlations between sST2 and (a) TBIL, (b) INR, and (c) MELD score.

Because the plasma level of sST2 increased after week 1 in patients who died during follow-up, we evaluated whether plasma sST2 level correlated with disease severity. Results of Spearman's rank correlation test showed that plasma levels of sST2 were correlated with TBIL, INR, and MELD scores in week 2, and these correlations became stronger in week 3 (Table 3, Figure 6).

### 3.5. Predictive Value of sST2 for Survival in Patients with HBV-ACLF

**Figure 7:**
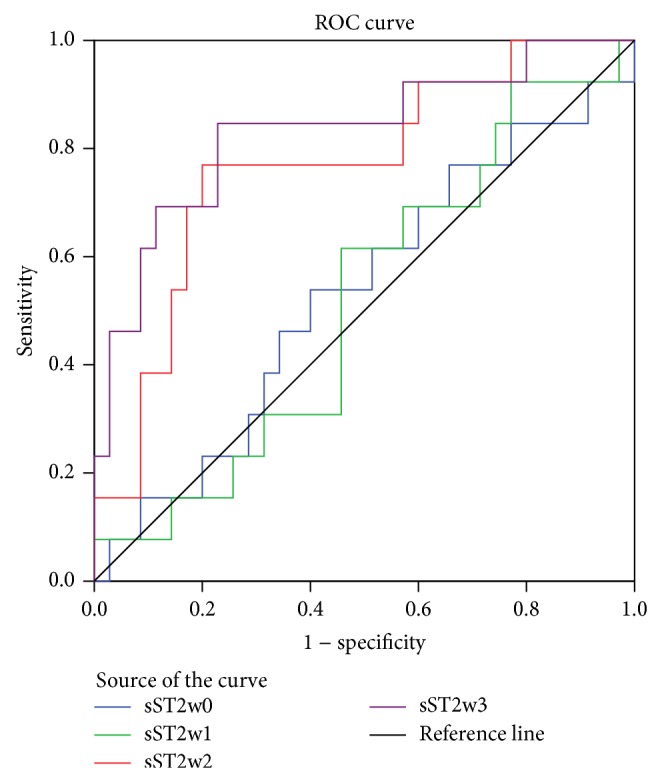
Receiver operating characteristic (ROC) curves to determine the ability of soluble ST2 (sST2) to predict survival for patients with hepatitis B virus-related acute-on-chronic liver failure. sST2w0, sST2 at week 0; sST2w1, sST2 at week 1; sST2w2, sST2 at week 2; sST2w3, sST2 at week 3.

**Figure 8:**
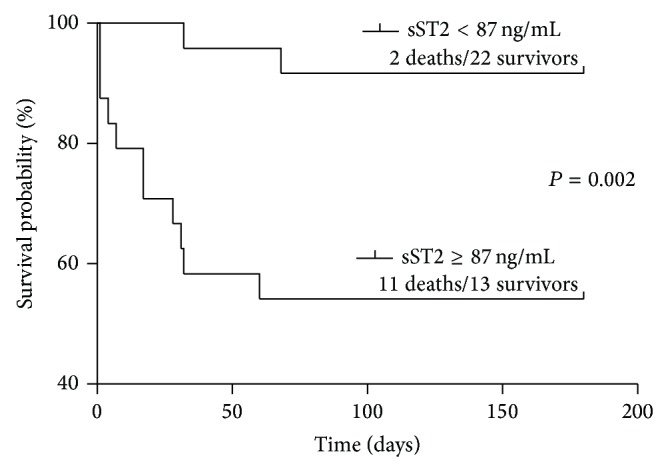
Kaplan-Meier survival curve showing survival probability of 48 patients with hepatitis B virus-related acute-on-chronic liver failure stratified into two groups according to plasma sST2 concentration at week 3.

The ability of sST2 to predict survival in patients with HBV-ACLF was evaluated by generating ROC curves and calculating sensitivity and specificity for different cut-off levels. We observed that the area under the curve (AUC) increased from week 0 to week 3 (Figure 7, Table 4). At week 3, the optimal cut-off value was 107.548 ng/mL, with a sensitivity and specificity of 84.6% and 76.5%, respectively (*P* < 0.001). We stratified the 48 patients with HBV-ACLF into two groups according to plasma concentration of sST2 at week 3 (<87 ng/mL, *n* = 24; ≥87 ng/mL, *n* = 24). Results of Kaplan-Meier analysis demonstrated that mortality was significantly higher among patients with higher sST2 plasma concentrations (log-rank test for trend, *P* = 0.002) (Figure 8).

## 4. Discussion

Previous studies have shown that IL-33 functions as an alarmin that is released following cell necrosis to alert the immune system to tissue damage or stress [15]. Our study was the first to demonstrate that plasma IL-33 levels are very low or undetectable in HBV-ACLF patients. In contrast with results of a study by Roth et al. [13], we did not observe a correlation between plasma IL-33 level and ALT or other liver function parameters; these conflicting results may be due in part to different assays and causes of liver damage.

Immunologically mediated events play an important role in the pathogenesis of HBV-related liver failure [34]. Fas-induced cell apoptosis leads to removal of HBV-infected hepatocytes by natural killer cells, natural killer T cells, and T lymphocytes [35]. Massive apoptosis in a short time leads to acute liver disease. During apoptosis, executioner caspases (caspase-3 and -7) inactivate IL-33 by cleaving the carboxy-terminal IL-1-like *β*-trefoil domain, and the cleaved IL-33 is largely retained inside apoptotic bodies [15, 17]. This is consistent with our finding that few IL-33^+^ cells were detected in liver tissues of HBV-ACLF patients.

IL-33 is expressed primarily in epithelial and endothelial cells, especially in high endothelial venules [14], and can enhance CD8^+^ T cell responses to infection [36], which serve as the first line of defense against microbes in Th2-type immune responses [14, 17]. Miyagaki et al. reported that low IL-33 levels are associated with an increased risk of opportunistic infections in patients with HIV infection [37], providing additional evidence that low IL-33 levels reflect impaired immunity. In addition, Volarevic et al. [26] showed that pretreatment with IL-33 prevented Concanavalin A-induced liver damage by preventing apoptosis of hepatocytes and Th2 amplification in mice. However, the potential role of IL-33/ST2 signaling in HBV-ACLF in humans requires further research.

Soluble ST2 acts as a decoy receptor for IL-33 [38], attenuating Th2 inflammatory responses by preventing IL-33 binding to transmembrane ST2, which is involved in several T cell-mediated diseases [23]. Previous studies have concluded that sST2 may be a useful biomarker for cardiovascular disease and inflammation, such as heart failure and sepsis [24, 28, 39]. In our study, plasma sST2 concentration was considerably higher in HBV-ACLF patients than in CHB patients and healthy controls but was not correlated with plasma IL-33 concentration. In addition, expression of ST2 protein was stronger in liver tissues of HBV-ACLF patients than in liver tissues of CHB patients and was associated with immune infiltrate.

Based on these results we focused on the role of sST2 in the pathogenesis of HBV-ACLF. Pan et al. previously reported that lipopolysaccharide (LPS) levels were significantly elevated in the peak phase of HBV-ACLF [40]. In addition, Han proposed that LPS plays an important role in liver failure induced by pathogenic factors (e.g., hepatitis B virus) as a secondary liver injury [41]. However, Sweet et al. reported that LPS activates macrophages via toll-like receptor (TLR) 4, inducing proinflammatory cytokines such as IL-1*β* and TNF-*α*, which activate fibroblasts and other cell types to produce sST2. The sST2 binds to macrophages and represses the expression of proinflammatory cytokines (e.g., IL-6, IL-12, TNF-*α*), possibly by downregulating TLR 4 [42]. Therefore, sST2 may function as a negative regulator in HBV-ACLF as in other inflammation diseases, which is upregulated by proinflammatory cytokines and LPS [43, 44]. Moreover, overexpression of ST2 had been reported to act as an effective negative-feedback role in selective TLR signalling, including contributing to endotoxin tolerance and inhibiting TH1-cell responses [45, 46]. In the current study, high levels of sST2 may imply a shift toward an anti-inflammatory immunosuppressive state in HBV-ACLF patients [47]. As suggested by Wasmuth et al. [27], patients with ACLF and severe sepsis show a similar degree of cellular immune depression. However, no correlation was found between plasma levels of sST2 and proinflammatory cytokines TNF-*α*, IFN-*γ* or the anti-inflammatory cytokine IL-10 in our study; thus further research is necessary to elucidate the exact source and function of sST2 in HBV-ACLF.

This study has several limitations. First, we did not measure LPS concentration in HBV-ACLF patients because some patients received antibiotics, which can alter serum LPS level [40, 48]. Second, the first blood specimen was obtained on the day of hospital admission rather than the onset of liver failure; therefore, patients may have been in different phases of ACLF during the first few weeks of the study during which blood specimens were obtained. Nevertheless, our data demonstrated that changes in sST2 predict outcome in HBV-ACLF, with a sustained elevation of sST2 associated with death. In addition, severe patients who died within three weeks since hospital admission were excluded from the current study, so the mortality of the patients included seems to be significantly lower than the mortality reported previously [5, 6].

To our knowledge, this is the first study to investigate the dynamic changes of sST2 in HBV-ACLF patients and determine the relationship between sST2 concentration and outcome. Our results showed that plasma sST2 concentration in weeks 2 and 3 correlated with liver function parameters reflecting disease severity (TBIL and INR) [4] and MELD scores. Similarly, ROC curve analysis showed that the ability of sST2 to predict outcome increased from week 1 to week 3, and Kaplan-Meier curve analyses demonstrated that higher plasma levels of sST2 at week 3 were predictive of mortality. These results strongly suggest that plasma sST2 level is associated with disease severity in HBV-ACLF, and a steady increase in sST2 may predict poor prognosis in these patients.

## 5. Conclusion

Our data showed that patients with HBV-ACLF had high plasma levels of sST2 and increased expression of ST2 protein associated with inflammatory infiltrates in liver tissue. Moreover, changes in plasma sST2 levels during the first 3 weeks correlated with mortality during the 180-day follow-up. These findings suggest that plasma sST2 level may be a potential biomarker of disease severity that could be used in making treatment decisions. More detailed studies are warranted to better understand the role of sST2 and IL-33 in the pathogenesis of HBV-ACLF.

## Figures and Tables

**Table 1:** Baseline characteristics of patients with hepatitis B-related acute-on-chronic liver failure (HBV-ACLF), patients with chronic hepatitis B, and healthy controls.

Characteristics	HBV-ACLF (*n* = 48)	Chronic hepatitis B (*n* = 12)	Healthy controls (*n* = 16)	Statistic	*P* value
Sex, male : female	45 : 3	11 : 1	12 : 4	*F* = 4.55	0.12
Age, years	38.38 ± 9.47	34.00 ± 7.80	36.38 ± 13.55	*F* = 0.95	0.39
ALT, U/L (median, IQR)	404.00 (139.75–815.25)	179.5 (70.75–246.00)	17.5 (16–22)	*H* = 42.51	<0.001
TBIL, *μ*mol/L (mean ± SD)	367.56 ± 190.58	23.26 ± 13.27	11.78 ± 3.5	*F* = 46.51	<0.001
INR (median, IQR)	1.89 (1.63–2.56)	0.98 (0.86–1.04)	0.93 (0.85–1.02)	*H* = 52.41	<0.001
HBV DNA, log⁡10 IU/mL, (mean ± SD)	5.96 ± 1.95	6.58 ± 1.26	NA	*t* = −1.05	0.29

ALT, alanine aminotransferase; INR, prothrombin time international normalized ratio; IQR, 25%–75% interquartile range; NA, not applicable; TBIL, total bilirubin. *P*-values for differences between three groups were determined with Student's *t*-test for parameter data and Kruskal-Wallis test for nonparametric data.

**Table 2:** Baseline characteristics of HBV-ACLF patients who survived during the 180-day follow-up and those who died.

Characteristics	Survived (*n* = 35)	Died (*n* = 13)	Statistic	*P* value
Sex, male : female	33 : 2	12 : 1		
Age, years	37.91 ± 9.00	39.62 ± 10.93	*t* = −0.549	0.586
ALT, U/L (median, IQR)	531.00 (266.00–1262.00)	92.00 (67.00–393.00)	*Z* = −3.631	0.000
TBIL, umol/L (*X* ± SD)	317.29 ± 169.24	502.92 ± 184.08	*t* = −3.312	0.002
INR (median, IQR)	1.78 (1.57–2.00)	3.00 (2.32–4.30)	*Z* = −3.666	0.000
HBV DNA (log_10_⁡ IU/mL, *X* ± SD)	5.85 (4.85–8.20)	5.73 (4.02–6.28)	*Z* = −1.090	0.272

ALT, alanine aminotransferase; INR, prothrombin time international normalized ratio; IQR, 25%–75% interquartile range; NA, not applicable; TBIL, total bilirubin. *P*-values for differences between two groups were determined with Student's *t*-test for parameter data and Mann-Whitney test for nonparametric data.

**Table 3:** Correlations between soluble ST2 (sST2) and clinical markers of disease severity in patients with hepatitis B virus-related acute-on-chronic liver failure.

	sST2			
	Week 2		Week 3	
	*r*	*P* value	*r*	*P* value
ALT	0.176	0.233	0.101	0.500
TBIL	0.616	<0.001	0.751	<0.001
INR	0.457	0.001	0.629	<0.001
MELD score	0.554	<0.001	0.735	<0.001

*P* values from Spearman's rank correlation test.

ALT, alanine aminotransferase; TBIL, total bilirubin; INR, prothrombin time international normalized ratio; MELD, Model for End-stage Liver Disease.

**Table 4:** Area under the curve (AUC), sensitivity, and specificity of the receiver operating characteristic curve to determine the ability of plasma soluble (sST2) concentration to predict survival in patients with hepatitis B virus-related acute-on-chronic liver failure.

Variables	AUC value (95% CI)	Cut-off value	Sensitivity	Specificity	*P* value
sST2w0	0.538 (0.350–0.727)	83.608	0.769	0.343	0.686
sST2w1	0.514 (0.335–0.693)	103.730	0.615	0.543	0.693
sST2w2	0.767 (0.613–0.921)	110.639	0.769	0.800	0.005
sST2w3	0.826 (0.688–0.973)	107.548	0.846	0.765	0.001

sST2w0, sST2 at week 0; sST2w1, sST2 at week 1; sST2w2, sST2 at week 2; sST2w3, sST2 at week 3.

## References

[1] Kohrt H. E., Ouyang D. L., Keeffe E. B. (2007). Antiviral prophylaxis for chemotherapy-induced reactivation of chronic hepatitis B virus infection. *Clinics in Liver Disease*.

[2] Millonig G., Kern M., Ludwiczek O., Nachbaur K., Vogel W. (2006). Subfulminant hepatitis B after infliximab in Crohn's disease: need for HBV-screening?. *World Journal of Gastroenterology*.

[3] Sheng W.-H., Kao J.-H., Chen P.-J. (2007). Evolution of hepatitis B serological markers in HIV-infected patients receiving highly active antiretroviral therapy. *Clinical Infectious Diseases*.

[4] Sarin S. K., Kumar A., Almeida J. A. (2009). Acute-on-chronic liver failure: consensus recommendations of the Asian Pacific Association for the study of the liver (APASL). *Hepatology International*.

[5] Miyake Y., Iwasaki Y., Takaki A. (2008). Lamivudine treatment improves the prognosis of fulminant hepatitis B. *Internal Medicine*.

[6] Neuberger J. (2005). Prediction of survival for patients with fulminant hepatic failure. *Hepatology*.

[7] Merion R. M., Sharma P., Mathur A. K., Schaubel D. E. (2011). Evidence-based development of liver allocation: a review. *Transplant International*.

[8] Kamath P. S., Kim W. R. (2007). The model for end-stage liver disease (MELD). *Hepatology*.

[9] Ambrosino G., Naso A., Feltracco P. (2003). Cytokines and liver failure: modification of TNF- and IL-6 in patients with acute on chronic liver decompensation treated with Molecular Adsorbent Recycling System (MARS). *Acta Biomedica*.

[10] Galun E., Axelrod J. H. (2002). The role of cytokines in liver failure and regeneration: potential new molecular therapies. *Biochimica et Biophysica Acta—Molecular Cell Research*.

[11] Zou Z., Li B., Xu D. (2009). Imbalanced intrahepatic cytokine expression of interferon-gamma, tumor necrosis factor-alpha, and interleukin-10 in patients with acute-on-chronic liver failure associated with hepatitis B virus infection. *Journal of Clinical Gastroenterology*.

[12] Masubuchi Y., Sugiyama S., Horie T. (2009). Th1/Th2 cytokine balance as a determinant of acetaminophen-induced liver injury. *Chemico-Biological Interactions*.

[13] Roth G. A., Zimmermann M., Lubsczyk B. A. (2010). Up-regulation of interleukin 33 and soluble ST2 serum levels in liver failure. *Journal of Surgical Research*.

[14] Moussion C., Ortega N., Girard J.-P. (2008). The IL-1-like cytokine IL-33 is constitutively expressed in the nucleus of endothelial cells and epithelial cells in vivo: a novel ‘Alarmin’?. *PLoS ONE*.

[15] Lamkanfi M., Dixit V. M. (2009). IL-33 raises alarm. *Immunity*.

[16] Belpinati F., Malerba G., Trabetti E. (2011). Association of childhood allergic asthma with markers flanking the IL33 gene in Italian families. *Journal of Allergy and Clinical Immunology*.

[17] Milovanovic M., Volarevic V., Radosavljevic G. (2012). IL-33/ST2 axis in inflammation and immunopathology. *Immunologic Research*.

[18] Liew F. Y. (2012). IL-33: a Janus cytokine. *Annals of the Rheumatic Diseases*.

[19] Yang Z., Liang Y., Xi W., Li C., Zhong R. (2011). Association of increased serum IL-33 levels with clinical and laboratory characteristics of systemic lupus erythematosus in Chinese population. *Clinical and Experimental Medicine*.

[20] Miller A. M. (2011). Role of IL-33 in inflammation and disease. *Journal of Inflammation*.

[21] Tominaga S. (1989). A putative protein of a growth specific cDNA from BALB/c-3T3 cells is highly similar to the extracellular portion of mouse interleukin 1 receptor. *FEBS Letters*.

[22] Schmitz J., Owyang A., Oldham E. (2005). IL-33, an interleukin-1-like cytokine that signals via the IL-1 receptor-related protein ST2 and induces T helper type 2-associated cytokines. *Immunity*.

[23] Kumar R. K., Foster P. S. (2002). ST2: marker, activator and regulator of Th2 immunity?. *Clinical and Experimental Allergy*.

[24] Bhardwaj A., Januzzi J. L. (2010). ST2: a novel biomarker for heart failure. *Expert Review of Molecular Diagnostics*.

[25] Mueller T., Dieplinger B., Gegenhuber A., Poelz W., Pacher R., Haltmayer M. (2008). Increased plasma concentrations of soluble ST2 are predictive for 1-year mortality in patients with acute destabilized heart failure. *Clinical Chemistry*.

[26] Volarevic V., Mitrovic M., Milovanovic M. (2012). Protective role of IL-33/ST2 axis in Con A-induced hepatitis. *Journal of Hepatology*.

[27] Wasmuth H. E., Kunz D., Yagmur E. (2005). Patients with acute on chronic liver failure display ‘sepsis-like’ immune paralysis. *Journal of Hepatology*.

[28] Hoogerwerf J. J., Tanck M. W. T., van Zoelen M. A. D., Wittebole X., Laterre P.-F., van der Poll T. (2010). Soluble ST2 plasma concentrations predict mortality in severe sepsis. *Intensive Care Medicine*.

[29] Liver Failure and Artificial Liver Group (2013). Diagnostic and treatment guidelines for liver failure (2012 version). *Zhonghua Gan Zang Bing Za Zhi*.

[30] Codices V., Martins C., Novo C. (2013). Dynamics of cytokines and immunoglobulins serum profiles in primary and secondary Cryptosporidium parvum infection: usefulness of Luminex xMAP technology. *Experimental Parasitology*.

[31] Jia Y., Hu D.-N., Zhou J. (2014). Human aqueous humor levels of TGF-beta 2: relationship with axial length. *BioMed Research International*.

[32] Marvie P., Lisbonne M., L'Helgoualc'h A. (2010). Interleukin-33 overexpression is associated with liver fibrosis in mice and humans. *Journal of Cellular and Molecular Medicine*.

[33] Arshad M. I., Rauch M., L'Helgoualc'h A. (2011). NKT cells are required to induce high IL-33 expression in hepatocytes during ConA-induced acute hepatitis. *European Journal of Immunology*.

[34] Guidotti L. G., Chisari F. V. (2006). Immunobiology and pathogenesis of viral hepatitis. *Annual Review of Pathology*.

[35] Malhi H., Gores G. J. (2008). Cellular and molecular mechanisms of liver injury. *Gastroenterology*.

[36] Bonilla W. V., Fröhlich A., Senn K. (2012). The alarmin interleukin-33 drives protective antiviral CD8^+^ T cell responses. *Science*.

[37] Miyagaki T., Sugaya M., Yokobayashi H. (2011). High levels of soluble ST2 and low levels of IL-33 in sera of patients with HIV infection. *Journal of Investigative Dermatology*.

[38] Oshikawa K., Yanagisawa K., Tominaga S., Sugiyama Y. (2002). Expression and function of the ST2 gene in a murine model of allergic airway inflammation. *Clinical and Experimental Allergy*.

[39] Chen L. Q., de Lemos J. A., Das S. R., Ayers C. R., Rohatgi A. (2013). Soluble ST2 is associated with all-cause and cardiovascular mortality in a population-based cohort: the Dallas Heart Study. *Clinical Chemistry*.

[40] Pan C., Gu Y., Zhang W. (2012). Dynamic changes of lipopolysaccharide levels in different phases of acute on chronic hepatitis B liver failure. *PLoS ONE*.

[41] Han D.-W. (2002). Intestinal endotoxemia as a pathogenetic mechanism in liver failure. *World Journal of Gastroenterology*.

[42] Sweet M. J., Leung B. P., Kang D. (2001). A novel pathway regulating lipopolysaccharide-induced shock by ST2/T1 via inhibition of toll-like receptor 4 expression. *The Journal of Immunology*.

[43] Kumar S., Tzimas M. N., Griswold D. E., Young P. R. (1997). Expression of ST2, an interleukin-1 receptor homologue, is induced by proinflammatory stimuli. *Biochemical and Biophysical Research Communications*.

[44] Saccani S., Polentarutti N., Penton-Rol G., Sims J. E., Mantovani A. (1998). Divergent effects of LPS on expression of IL-1 receptor family members in mononuclear phagocytes in vitro and in vivo. *Cytokine*.

[45] Brint E. K., Xu D., Liu H. (2004). ST2 is an inhibitor of interleukin 1 receptor and Toll-like receptor 4 signaling and maintains endotoxin tolerance. *Nature Immunology*.

[46] Liew F. Y., Xu D., Brint E. K., O'Neill L. A. J. (2005). Negative regulation of toll-like receptor-mediated immune responses. *Nature Reviews Immunology*.

[47] Xing T., Li L., Cao H., Huang J. (2007). Altered immune function of monocytes in different stages of patients with acute on chronic liver failure. *Clinical and Experimental Immunology*.

[48] Sandler N. G., Koh C., Roque A. (2011). Host response to translocated microbial products predicts outcomes of patients with HBV or HCV infection. *Gastroenterology*.

